# Continuous Exposure to 1.7 GHz LTE Electromagnetic Fields Increases Intracellular Reactive Oxygen Species to Decrease Human Cell Proliferation and Induce Senescence

**DOI:** 10.1038/s41598-020-65732-4

**Published:** 2020-06-08

**Authors:** Jisu Choi, Kyeongrae Min, Sangbong Jeon, Nam Kim, Jeong-Ki Pack, Kiwon Song

**Affiliations:** 1 Department of Biochemistry, College of Life Science & Biotechnology, Yonsei University, Seoul, 03722 Korea; 2 Radio & Satellite Research Division, Broadcasting·Media Research Laboratory, Electronics and Telecommunications Research Institute (ETRI), Daejon, 34129 Korea; 3 School of Information and Communication Engineering, Chungbuk National University, Cheongju, Chungbuk 28644 Korea; 4 Department of Radio and Information Communications Engineering, Chungnam National University, Daejon, 34134 Korea

**Keywords:** Cell biology, Risk factors

## Abstract

Due to the rapid development of mobile phone technology, we are continuously exposed to 1.7 GHz LTE radio frequency electromagnetic fields (RF-EMFs), but their biological effects have not been clarified. Here, we investigated the non-thermal cellular effects of these RF-EMFs on human cells, including human adipose tissue-derived stem cells (ASCs), Huh7 and Hep3B liver cancer stem cells (CSCs), HeLa and SH-SY5Y cancer cells, and normal fibroblast IMR-90 cells. When continuously exposed to 1.7 GHz LTE RF-EMF for 72 h at 1 and 2 SAR, cell proliferation was consistently decreased in all the human cells. The anti-proliferative effect was higher at 2 SAR than 1 SAR and was less severe in ASCs. The exposure to RF-EMF for 72 h at 1 and 2 SAR did not induce DNA double strand breaks or apoptotic cell death, but did trigger a slight delay in the G1 to S cell cycle transition. Cell senescence was also clearly observed in ASC and Huh7 cells exposed to RF-EMF at 2 SAR for 72 h. Intracellular ROS increased in these cells and the treatment with an ROS scavenger recapitulated the anti-proliferative effect of RF-EMF. These observations strongly suggest that 1.7 GHz LTE RF-EMF decrease proliferation and increase senescence by increasing intracellular ROS in human cells.

## Introduction

The development of wireless communication technology has made our life efficient and convenient. In return, we are continuously exposed to radio frequency electromagnetic fields (RF-EMFs) and environmental exposure to RF-EMFs has steadily increased. The International Agency for Research on Cancer (IARC) classified radiofrequency electromagnetic fields as Group 2B carcinogens in 2011^1,2^. However, biological studies have not consistently supported or clarified the carcinogenic effect of RF-EMF. A review of 2012 suggested that the currently available data did not show genotoxic effect from RF-EMF^3^. Two recent animal studies by the US National Toxicology Program^4,5^ and the Ramazzini Institute^6^ investigated the carcinogenic potential of long-term exposure to RF-EMFs associated with mobile phones. The International Commission on Non-ionizing Radiation Protection (ICNRP) evaluated these two reports and recently announced that the conclusions concerning the carcinogenic potential of RF-EMFs could not be drawn due to the technical limitations of the studies^7^. On the other hand, a recent report showed that rats exposed from prenatal life until natural death to 1.8 GHz global system for mobile (GSM) communication increased the incidence of heart malignant schwannoma among males exposed at the highest dose^8^. Thus, the carcinogenic effects of RF-EMF are still unclear.

Currently, 1700 to 1950 MHz RF-EMFs are the most widely used frequencies in mobile communications. Several studies have shown that 1800 MHz RF-EMF did not induce DNA damage or abnormal cellular behaviors in human neurogenic cells, skin fibroblast, and hematopoietic stem cells^9–11^. On the other hand, some studies reported the adverse effect of 1800 MHz on mouse embryonic neural stem cells and alterations of the gene expression profile in rat neurons^12,13^. Radiation of 1800 MHz also induced damage in mouse immortalized germ cells and spermatozoa *in* vitro^14^. Exposure to 1800 MHz RF has been reported to induce oxidative damage in mitochondrial DNA and the cellular functions of cultured human neurogenic cells and lens epithelial cells^15,16^. These inconsistencies may be due to differences in exposure devices, exposure conditions, or the source of the cells. In addition, recent wireless communication technology is using 4^th^ generation communication long-term evolution (4G-LTE), which provides very fast internet speeds over currently used radio frequencies. However, the cellular effects of LTE RF-EMF on various human cells have not yet been well documented.

The physiological impact of RF on tissues or cells involves both thermal and non-thermal effects^17^. Studies on 900 MHz RF-EMF have proposed that heat, ROS generation, disruption of calcium homeostasis, and changes in gene expression are the major mechanisms involved in the biological effects of electromagnetic fields^18–21^.

In this study, we investigated the non-thermal effects of 1.7 GHz LTE RF-EMF on the growth of various human cells including adipose tissue-derived stem cells (ASCs), liver cancer stem cell (CSC) populations of Huh7 and Hep3B, the neuroblastoma SH-SY5Y, the cervical cancer HeLa, and the normal fibroblast IMR-90 cells. Considering the current maximum permitted exposure values (2 W/kg in Europe and 1.6 W/kg in the US)^22^, we tested the effect of 1.7 GHz LTE RF-EMF at 1 W/kg (SAR) and 2 W/kg.

## Results

### Continuous exposure to 1.7 GHz LTE RF-EMF decreased human cell proliferation

**Figure 1:**
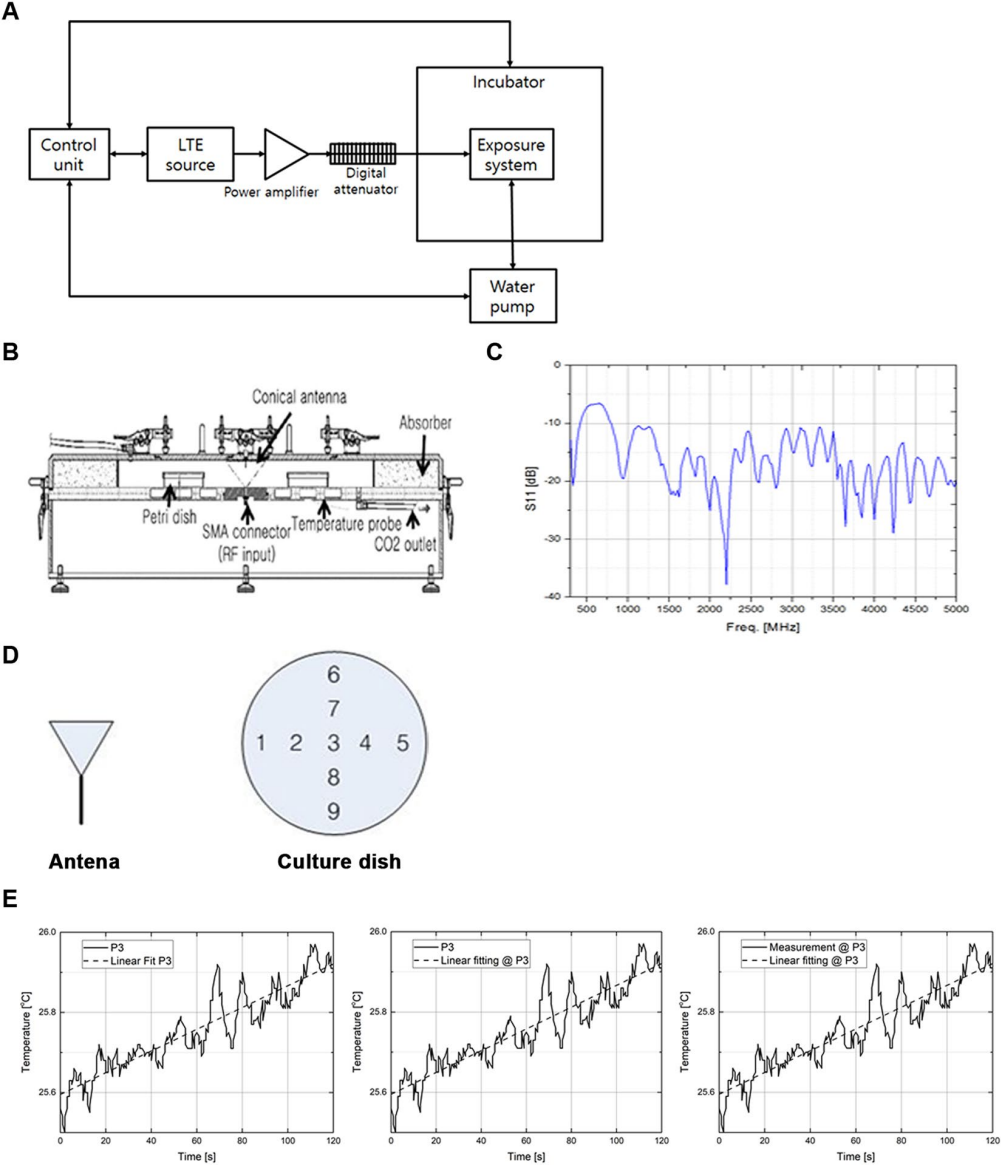
Design of the 1.7 GHz LTE RF-EMF cell exposure system. (**A**) A schematic diagram of the radial transmission line (RTL) exposure system. (**B**) Cross-sectional view of the RTL exposure chamber. (**C**) Return loss characteristics of the RTL exposure chamber. (**D**) Antenna and the measurement points in each culture plate. (**E**) Temperature and linear fitting for the center point at the LTE 1.7 GHz frequency. Temperature was measured without circulating water during RF exposure.

**Figure 2:**
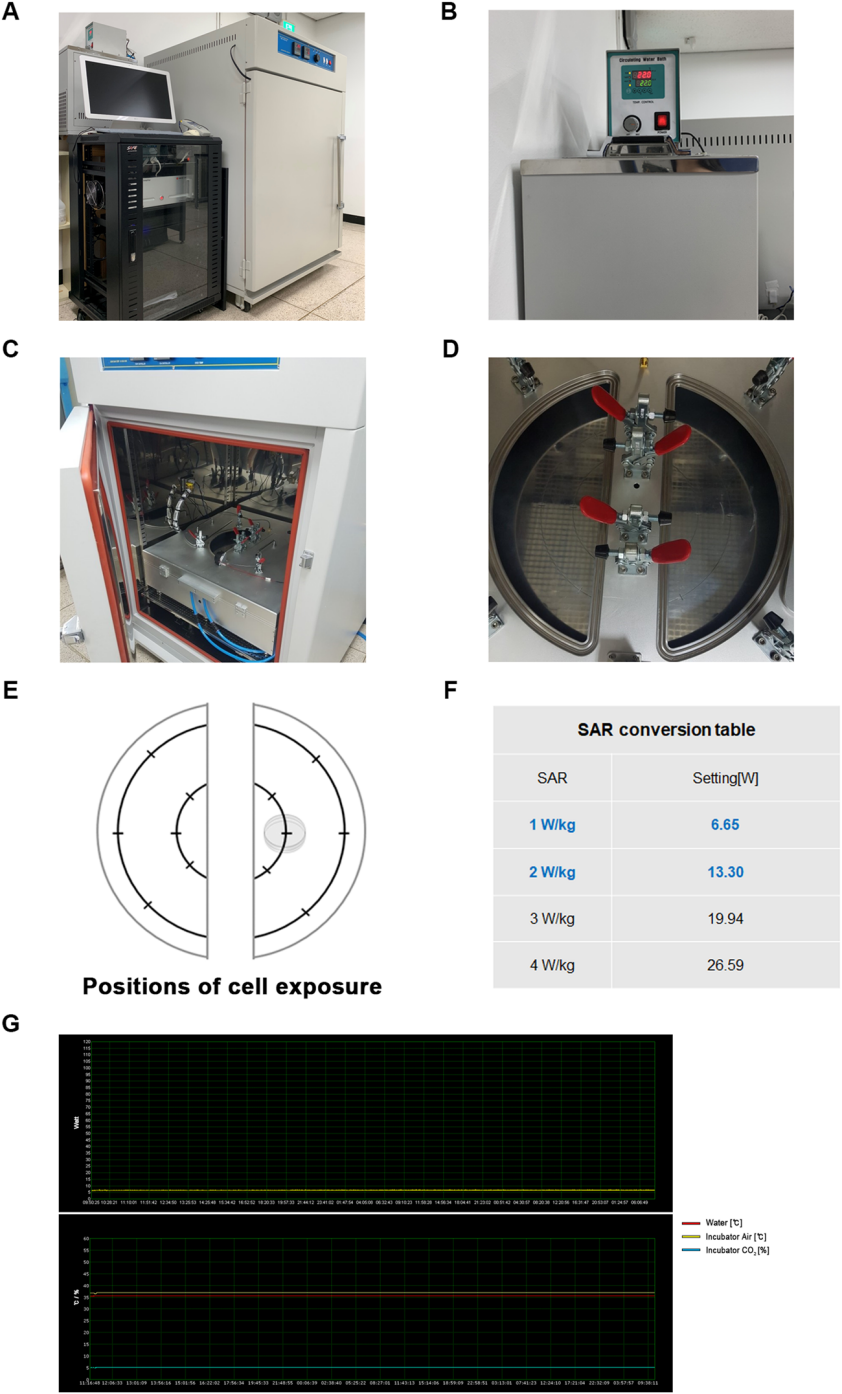
1.7 GHz LTE RF-EMF cell exposure device and its water cooling system. (**A**) The 1.7 GHz LTE RF-EMF cell exposure device used. (**B**) A water cooling system for the incubator to forcibly lower the heated water temperature by 1.7 GHz RF-EMF. (**C**) The chamber of the incubator with a 1.7 GHz RF-EMF LTE antenna. (**D**) A plate for cell culture dishes in (**C**) are located 13.6 cm from the conical antenna in the center of the exposure chamber. (**E**) A diagram of (**D**) designating the position of the cell dishes for accurate SAR exposure. (**F**) The SAR conversion table for this RF-EMF exposure device. SAR values for precise exposure conditions were obtained through engineering calculations. (**G**) The X-axis in the upper and lower graphs represents the real-time at which the RF-EMF is being exposed to cells. The Y-axis in the upper graph represents the SAR value (Watt) of RF-EMF during the exposure. The Y-axis in the bottom graph shows the temperature of the incubator (yellow line) and the temperature of the refrigerated water-cooling system (red line) of the RF-EMF exposure device during experiment.

Electro-magnetic exposure devices are not commercially standardized and are generally manufactured in various forms depending on the purpose of study^23^. We designed an RTL structured device in this study, and the detailed information on the device was described in Materials and Methods (Figs. 1 and 2). Our aim of this study was to investigate the non-thermal effect of 1.7 GHz LTE RF-EMF. Thus, we tried to minimize the thermal effect by installing a forced refrigerated water-cooling system in the incubator attached to the antenna generating 1.7 GHz LTE RF-EMF (Fig. 2). In order to investigate the non-thermal cellular effect of 1.7 GHz LTE RF-EMF on various human cells, we continuously incubated ASCs, a liver CSC population of Huh7 and Hep3B, HeLa and SH-SY5Y cancer cells, and normal fibroblast IMR-90 cells for 72 h in a 1.7 GHz LTE RF-EMF at 1 and 2 SAR, respectively.

**Figure 3:**
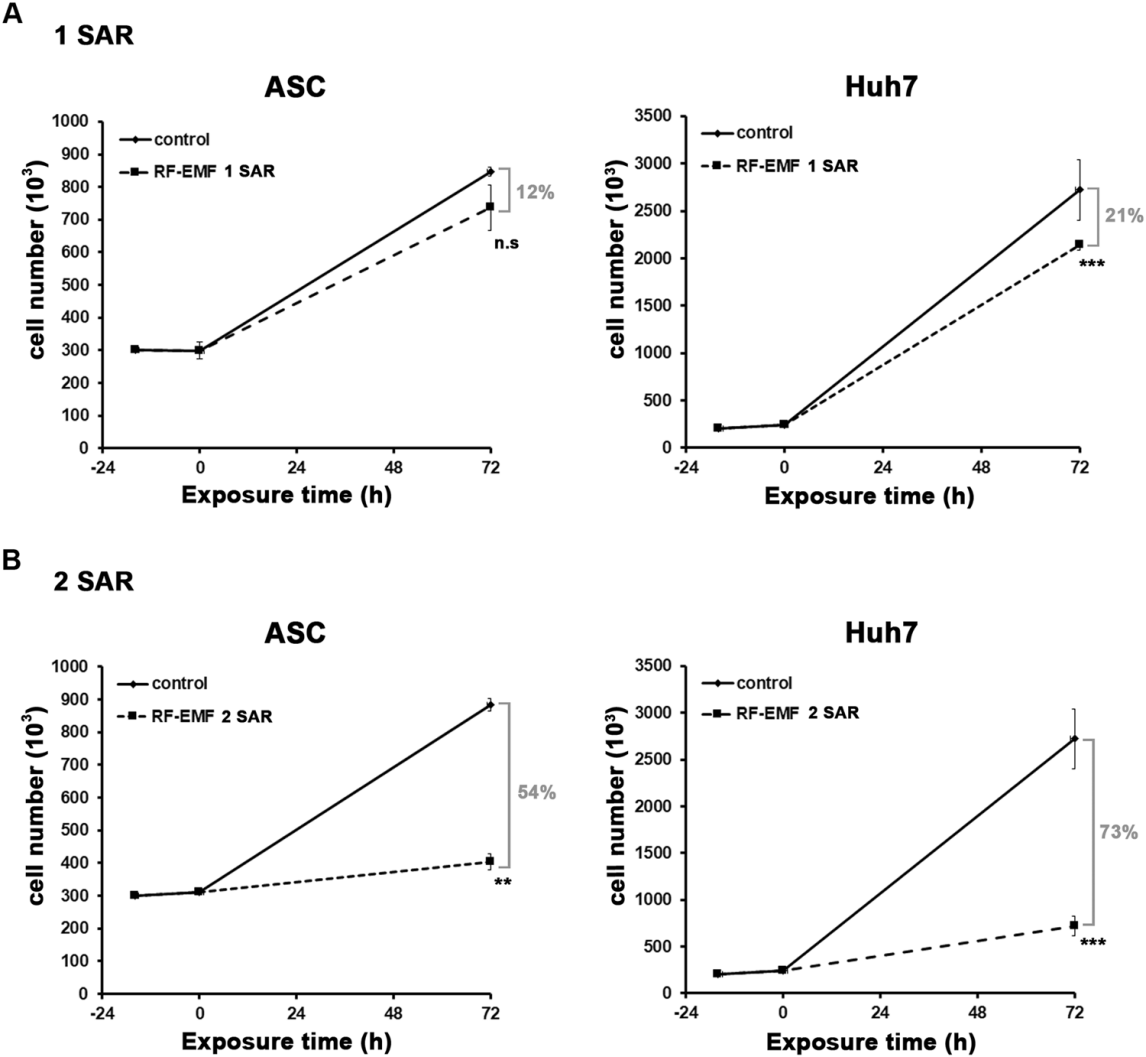
Continuous exposure to 1.7 GHz LTE RF-EMF decreased ASC and Huh7 cell proliferation. (**A,B**) ASCs and Huh7 cells prepared as described in Materials and Method were exposed to 1.7 GHz LTE RF-EMF for 72 h at 1 SAR (**A**) or 2 SAR (**B**). The sham control cells were incubated for 72 h without RF-EMF exposure. After the exposure, cells were collected, and counted with a cell counter (Nexcelom Bioscience). Three independent experiments were performed and the cell number was plotted as mean ± S.D. P < 0.01(**), P < 0.001(***), and P > 0.05 (non-significant, n.s.).

**Figure 4:**
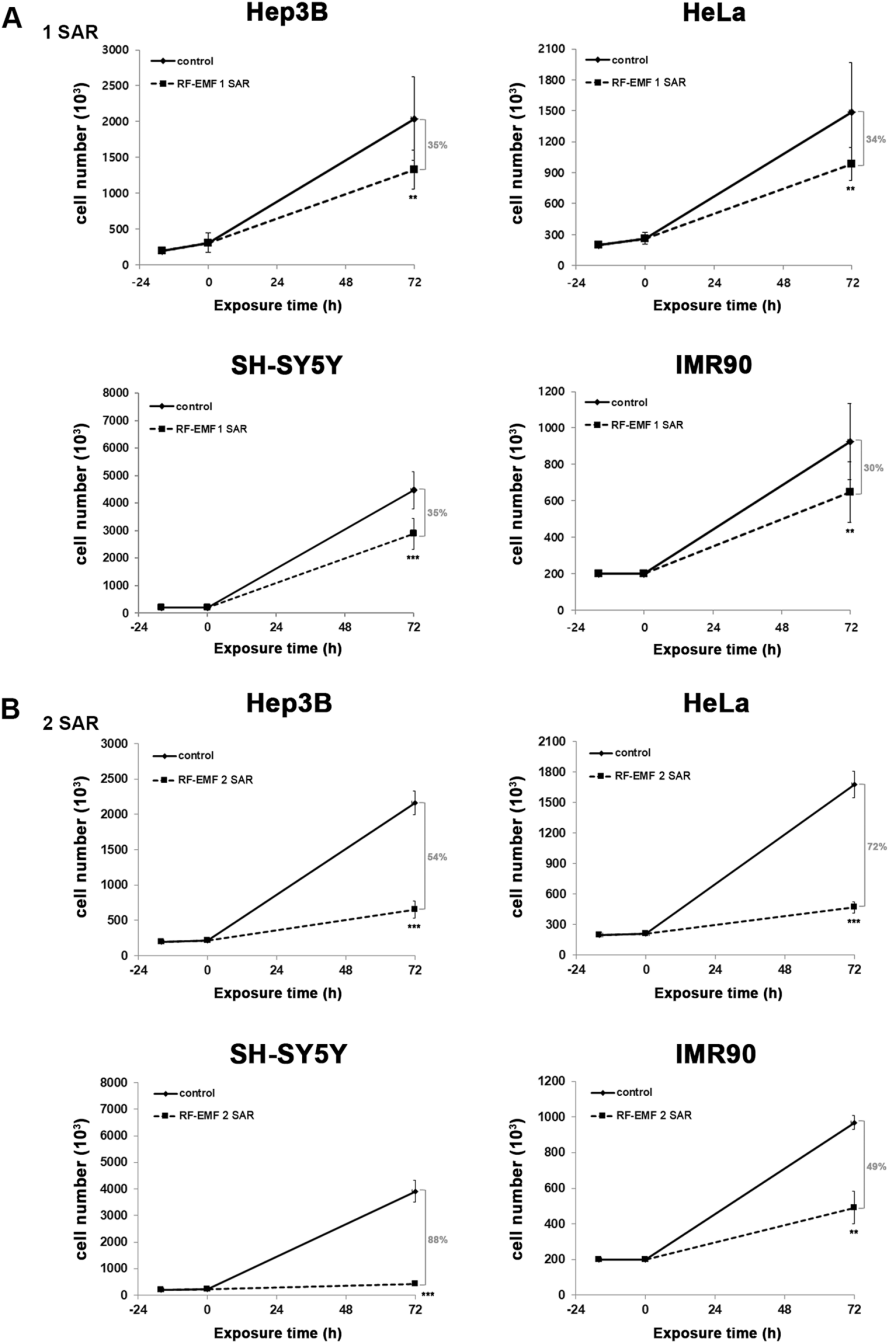
Continuous exposure to 1.7 GHz LTE RF-EMF decreased the proliferation of various human cells. (**A,B**) Hep3B, HeLa, SH-SY5Y, and IMR 90 cells were exposed to LTE 1.7 GHz RF-EMF for 72 h at 1 SAR (**A**) or 2 SAR (**B**). The sham control cells were incubated for 72 h without RF-EMF exposure. After the exposure, cells were collected and counted with a cell counter (Nexcelom Bioscience). At least three independent experiments were performed and the cell numbers were plotted as mean ± S.D. P < 0.01(**), P < 0.001(***), and P > 0.05 (non-significant, n.s).

When we first examined the cellular effect of 1.7 GHz LTE RF-EMF at 1 and 2 SAR on ASCs and Huh7, the cell proliferation of both ASC and Huh7 was decreased (Fig. 3A). Compared with the unexposed control, ASC proliferation decreased 12% at 1 SAR and 54% at 2 SAR (Fig. 3A,B). The anti-proliferative effect of 1.7 GHz LTE RF-EMF was more severe in Huh7 than ASCs: 21% at 1 SAR and 73% at 2 SAR (Fig. 3B). These results showed that the anti-proliferation effect of 1.7 GHz LTE RF-EMF was more serious at 2 SAR than at 1 SAR. To confirm the anti-proliferative effect of 1.7 GHz LTE RF-EMF to human cells in general, we also examined the proliferation of Hep3B cells, another type of liver CSC population, HeLa uterine cancer cells, neuroblastoma SH-SY5Y cells, and normal fibroblast IMR90 cells, after cells were exposed to 1.7 GHz LTE RF-EMF for 72 h at 1 SAR and 2 SAR. As with Huh7, proliferation of these cells all decreased by about 30~35% at 1 SAR and 49~88% at 2 SAR (Fig. 4). Interestingly, we noticed that neuroblastoma SH-SY5Y cells were notably more sensitive to 1.7 GHz LTE RF-EMF at 2 SAR than the other cells examined. These results demonstrated that continuous exposure to 1.7 GHz LTE RF-EMF for 72 h consistently decreased the proliferation of both cancer and normal cells regardless of their tissue of origin. In addition, ASCs were not as sensitive as other cancer and normal cells to 1.7 GHz LTE RF-EMF exposure.

### 1.7 GHz LTE RF-EMF did not induce DNA damage and apoptotic cell death

We especially focused on studying the mechanism of anti-proliferative effect in ASCs and a population of Huh7 CSCs because ASCs showed the least sensitive among examined human cells while Huh7 cells were highly sensitive to the 1.7 GHz LTE RF-EMF, as shown in Figs. 3–4. The key physiological functions of ASCs as mesenchymal lineage adult stem cells that can be easily isolated from the body and differentiated into various types of cells to be used in stem cell therapy also attracted us to study the effect of 1.7 GHz LTE RF-EMF on ASCs^20^. We also were interested in the anti-proliferative effect of 1.7 GHz LTE RF-EMF on Huh7 CSCs because CSCs play pivotal roles in cancer formation and recurrence^24–26^. Huh7 is a hepatocyte-derived carcinoma cell line and its stem cell populations are isolated using the markers, CD133 and epithelial cell adhesion molecule (EpCAM)^27,28^.

**Figure 5:**
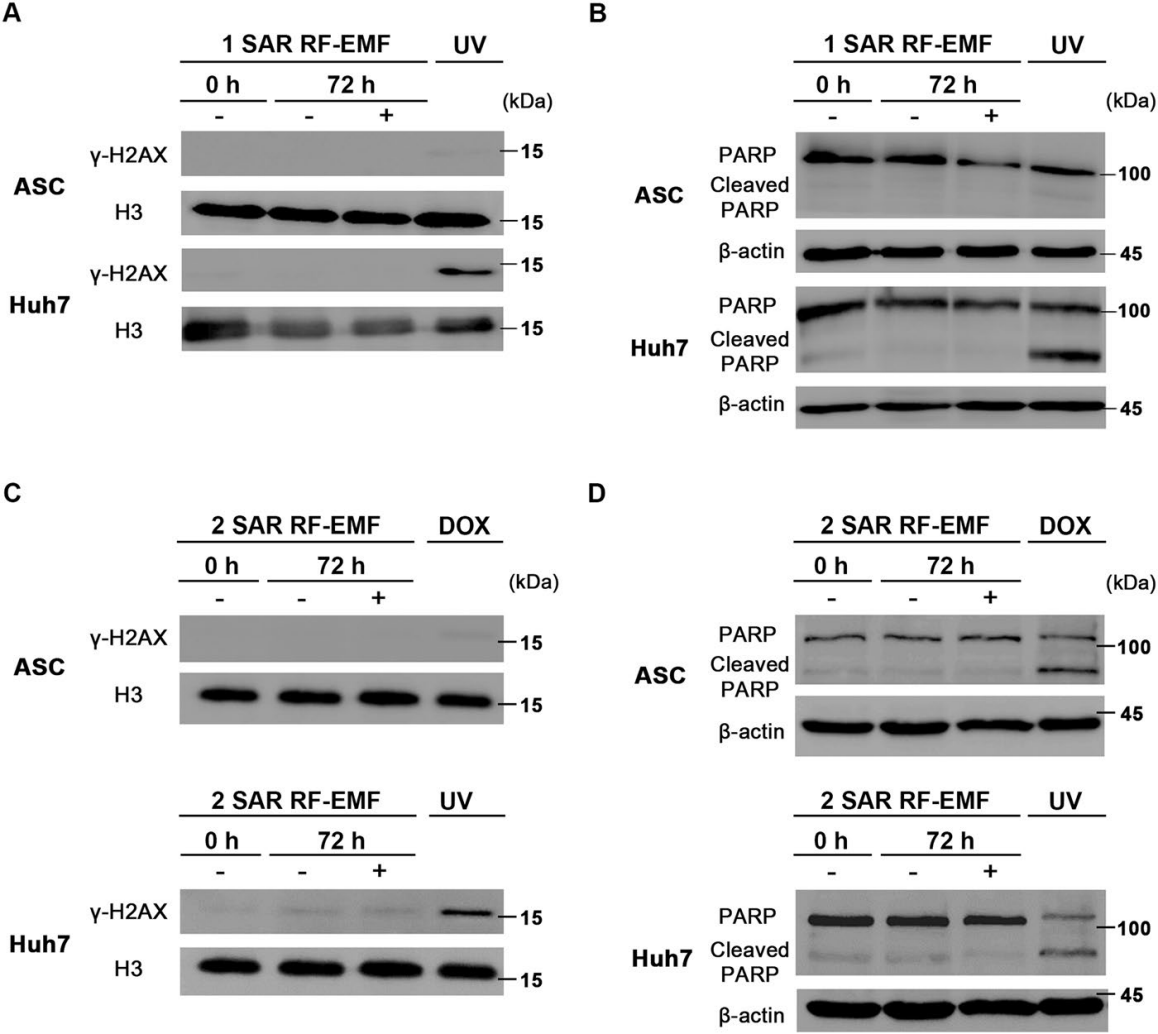
Continuous 1.7 GHz LTE RF-EMF exposure did not induce DNA damage and cellular apoptosis in ASCs and Huh7 cells. (**A–D**) ASCs and Huh7 cells were continuously exposed to 1.7 GHz LTE RF-EMF for 72 h at 1 SAR (**A,B**) or 2 SAR (**C,D**) and collected. Cells were lysed with lysis buffer and histones were extracted, which were separated by 8–15% SDS-polyacrylamide gel electrophoresis (PAGE) for western analyses. Western blots were performed with (**A,C**) anti γ-H2AX and anti-histone H3 antibodies, (**B,D**) anti-PARP and actin antibodies. Cells exposed to UV or treated with 3 μM doxorubicin (DOX) were used as positive controls for DNA damage and apoptosis. (**A,C**) Histone H3 and (**B,D**) β-actin from the same blot were used as a loading control. More than three independent replicates of all experiments were performed. The original full-length blots/gels were presented in the Supplementary Information.

In order to understand whether the decrease in cell proliferation caused by 1.7 GHz LTE RF-EMF was due to DNA damage and cell death, we examined whether 1.7 GHz LTE RF-EMF induces DNA double strand breaks (DSBs) and apoptosis in ASCs and Huh7. We detected the expression of phospho-histone 2AX (γ-H2AX) as a DNA DSB marker and cleaved poly ADP-ribose polymerase (PARP) as an apoptosis marker with western blots, after ASCs and Huh7 were exposed to 1.7 GHz LTE RF-EMF for 72 h at 1 SAR. ASCs and Huh7 cells treated with UV or doxorubicin were used as a control for DNA DSBs and apoptotic cell death. DNA DSBs and cleaved PARP were not observed in either ASCs or Huh7 exposed to 1.7 GHz LTE RF-EMF for 72 h at 1 SAR, while we could detect γ-H2AX and cleaved PARP in Huh 7 cells treated with UV (Fig. 5A,B). Considering that the anti-proliferative effect becomes more severe when cells were exposed to 2 SAR of 1.7 GHz LTE RF-EMF (Fig. 3), we also examined the expression of γ-H2AX and cleaved PARP at 2 SAR. However, γ-H2AX and cleaved PARP were not detected even in ASC and Huh7 cells exposed to 2 SAR (Fig. 5C,D). These observations strongly suggest that the decreased cell proliferation was caused neither by DNA damage nor apoptotic cell death. Interestingly, consistent with many reports that adult stem cells were not as sensitive to various stresses as other cells^29^, ASCs treated with UV did not show any γ-H2AX or cleaved PARP. ASCs showed faint γ-H2AX and cleaved PARP when treated with doxorubicin, which was used as a control.

### 1.7 GHz LTE RF-EMF decreased cell proliferation by inducing cell senescence

**Figure 6:**
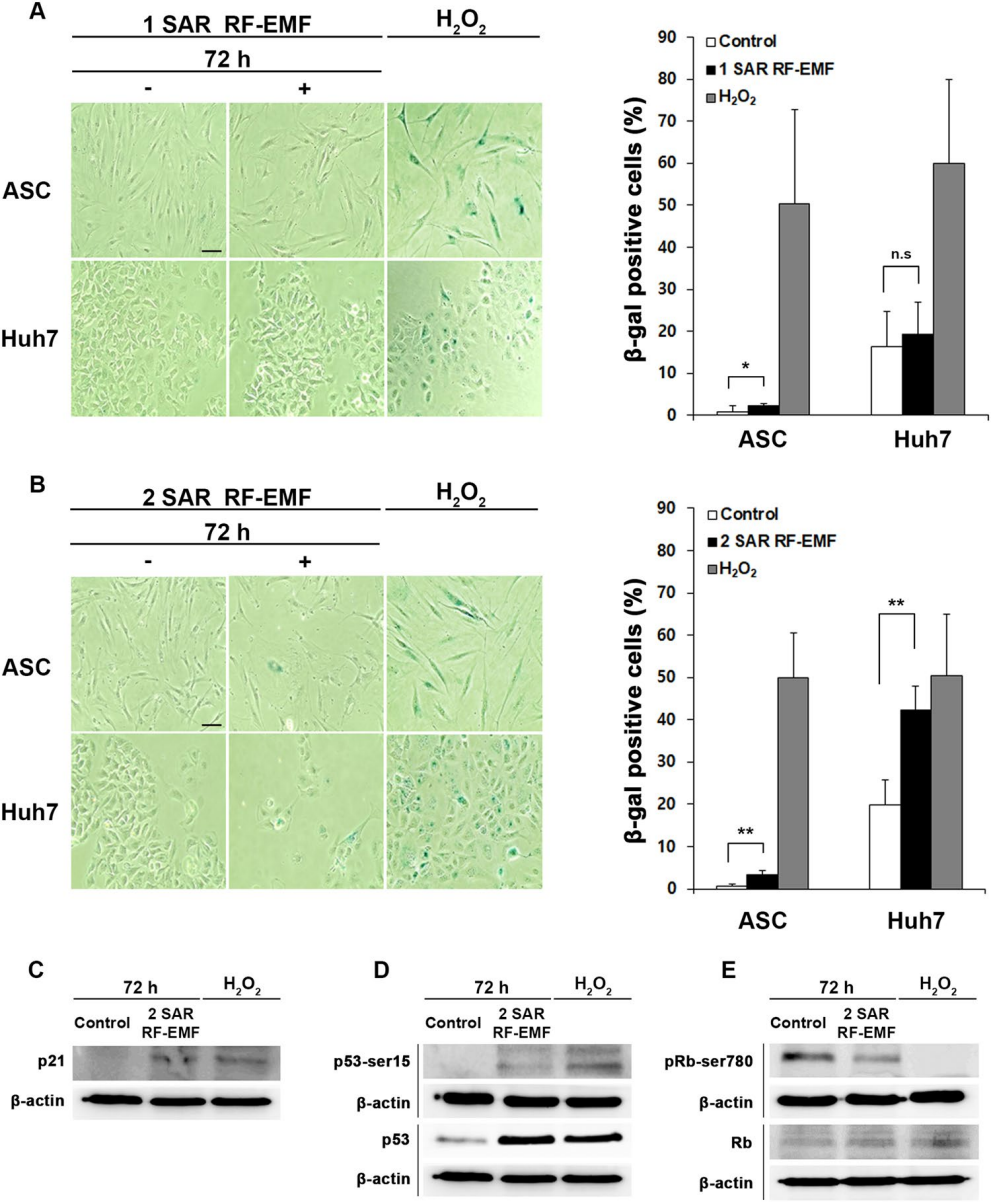
Continuous exposure to 1.7 GHz LTE RF-EMF induced cellular senescence in ASCs and Huh7 cells. (**A,B**) ASCs and Huh7 cells exposed to 1.7 GHz LTE RF-EMF for 72 h at 1 SAR (A) or 2 SAR (**B**). Cells were fixed in 2% formaldehyde and 0.2% glutaraldehyde, and incubated with 0.1% X-gal for 30 h. ASCs treated with 200 μM H_2_O_2_ for 1 h and Huh7 cells treated with 300 μM H_2_O_2_ for 2 h were respectively used as a positive control. Images were taken with a Nikon microscope (ECLIPSE Ts2) under a 10× objective. Scale bar, 50 μm. A total 200 cells were counted for each experiment. The percentages are SA-β-gal positive cells over total cells counted. The experiment was performed in triplicate and the cell percentage was plotted as mean ± S.D. P < 0.05 (*), P < 0.01(**) and P > 0.05 (non-significant, n.s.). (**C–E**) 1.7 GHz LTE RF-EMF exposed Huh7 cells (**B**) were subjected to western blots with (**C**) anti-p21, (**D**) anti-phosphorated-p53 at Ser15 and anti-p53, (**E**) anti-Rb and anti-phosphorylated-Rb at Ser 780. β-actin from the same blot was used as a loading control. More than three independent replicates were performed for all experiments. The original full-length blots were presented in the Supplementary Information.

After confirming that the decreased cell proliferation caused by 1.7 GHz LTE RF-EMF was not directly due to DNA damage and cell death, we examined whether the decline of cell proliferation would be due to cell senescence, which slows cell cycle progression and decreases cell growth. Therefore, we assayed senescence-associated (SA) β-galactosidase activity in ASCs and Huh7 cells exposed to 1.7 LTE GHz RF-EMF for 72 h at 1 and 2 SAR. ASCs and Huh7 cells treated with hydrogen peroxide (H_2_O_2_) were also stained with SA-β-gal as a positive control for cell senescence. Compared with the RF-untreated cells, LTE RF-EMF exposure at 1 SAR increased the number of cells stained with SA-β-gal by only 2% in ASCs and by 4% in Huh7, while H_2_O_2_ treatment increased the number of SA-β-gal-positive cells both in the ASC and Huh7 by more than 50% (Fig. 6A). When exposed to 2 SAR of 1.7 GHz LTE RF-EMF, SA-β-gal-stained cells were slightly increased to 3% in ASCs and were highly increased to 42% in Huh7 cells, compared with the untreated control (Fig. 6B). The increase in senescence-positive cells is consistent with the observation that the anti-proliferative effect of 2 SAR becomes more severe than that of 1 SAR and that Huh7 cells are more sensitive than ASCs.

We then detected molecular markers for cellular senescence in Huh7 cells exposed to 1.7 GHz LTE RF-EMF for 72 h at 2 SAR, since Huh7 cells were more sensitive than ASCs and the expression of senescence markers would be obvious. The augmented p21 expression, the increased phosphorylation of p53 at serine 15, and the decreased phosphorylation of retinoblastoma (Rb) at serine 780 have all been reported as markers for cell cycle arrest associated with senescence^30,31^. Thus, we examined the expression of these markers in Huh7 cells exposed to 1.7 GHz LTE RF-EMF for 72 h at 2 SAR. The expression of p21 was increased, while it was barely detected in the unexposed control cells (Fig. 6C). As with the case of hydrogen peroxide-treated positive control cells of senescence, the levels of Ser 15 phosphorylation as well as the total amount of p53 in exposed Huh7 cells were slightly higher than in those of the unexposed control (Fig. 6D). The expression of phosphorylated Rb at Ser 780 was strong in the unexposed control but was notably decreased in Huh7 cells exposed to 1.7 GHz LTE RF-EMF and was not detected in the hydrogen peroxide-treated positive control cells of senescence (Fig. 6E). The total amount of Rb in exposed Huh7 cells was similar with that in the positive and negative control cells (Fig. 6E). The expressions of these molecular markers for cellular senescence strongly supported the notion that exposure to 1.7 GHz LTE RF-EMF decreases the proliferation by inducing cell senescence.

### Continuous exposure to 1.7 GHz LTE RF-EMF delayed cell cycle progression at G1/S

**Figure 7:**
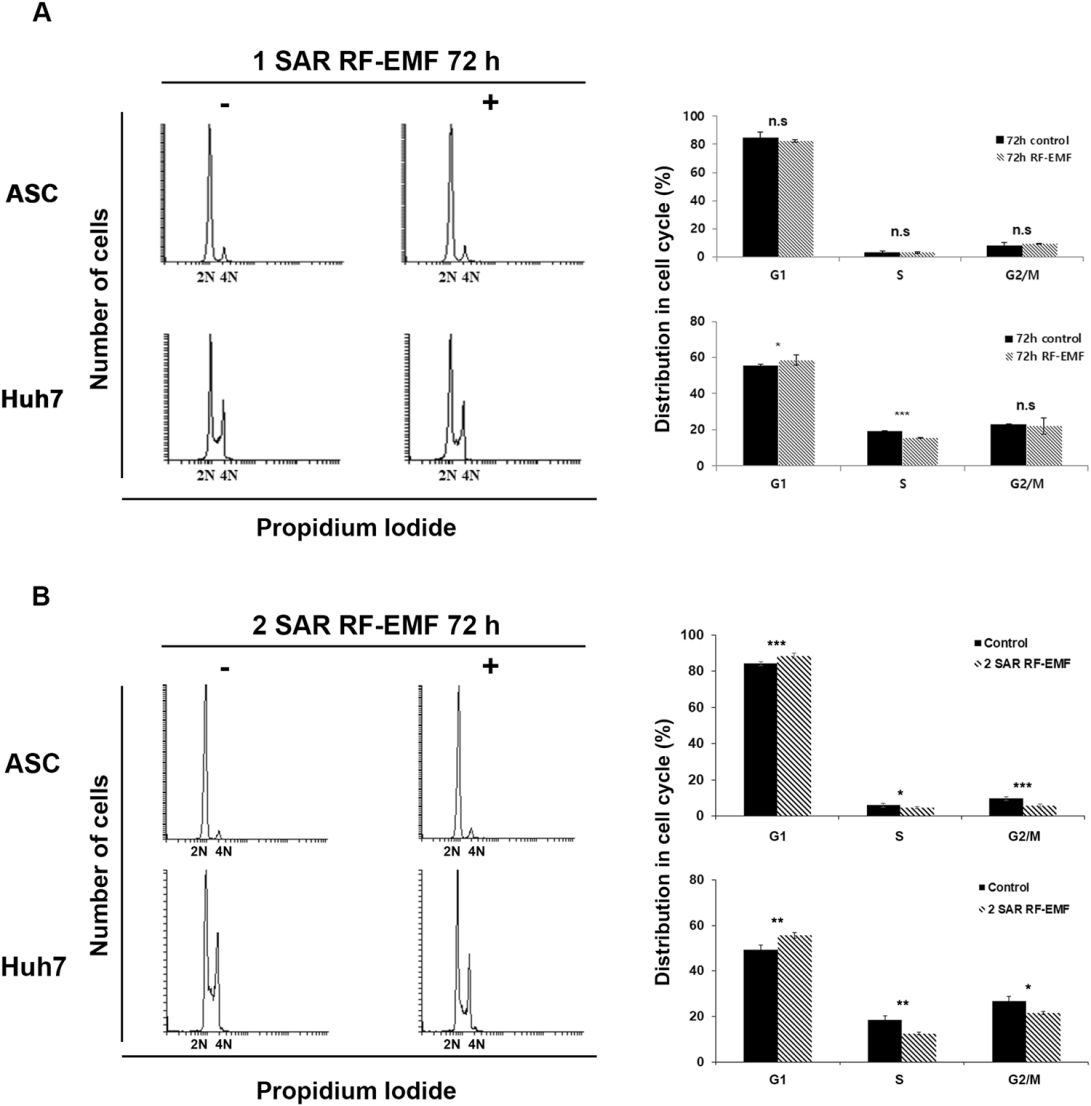
Continuous exposure to 1.7 GHz LTE RF-EMF induced cell cycle delay at G1 phase in ASCs and Huh7 cells. (**A–B**) ASC and Huh7 cells were exposed to 1.7 GHz RF-EMF for 72 h at 1 SAR (**A**) or 2 SAR (**B**). The DNA content of the 1.7 GHz RF-EMF-exposed and the unexposed sham control cells was analyzed by flow cytometry (BD Bioscience) after PI staining. 100,000 cells were counted for each experiment. The FACS results were analyzed using Flowing software 2 and the distribution of cells in each stage of the cell cycle was calculated from the FACS results shown in the left panel using Flowing software 2. P < 0.05 (*), P < 0.01(**), P < 0.001(***), and P > 0.05 (non-significant, n.s.).

We then examined the DNA content of ASCs and Huh7 cells exposed to 1.7 GHz LTE RF-EMF for 72 h by flow cytometry to confirm that the exposure did not induce cell death but cell senescence. Consistent with the western blot results shown in Fig. 5, no sub G1 population was detected in the ASCs or Huh7 cells at either 1 or 2 SAR, confirming that 1.7 GHz LTE RF-EMF did not induce cell death (Fig. 7). We also observed no meaningful change in the cell cycle of ASCs exposed to 1 SAR (Fig. 7A). Huh7 cells exposed to 1 SAR showed slightly increased G1 and decreased S populations in comparison with the cells of unexposed control (Fig. 7A). Interestingly, when exposed to 2 SAR of 1.7 GHz RF-EMF, we could clearly observe the delay of the cell cycle at G1/S progression in Huh7 cells: the G1 population was increased 6% and S population decreased 6% compared with the unexposed control (Fig. 7B). These observations were consistent with the activation of p21 and p53 in Huh7 cells exposed to 2 SAR of 1.7 GHz RF-EMF, as shown in Fig. 6C,D. In addition, we could also observe a slight delay in the cell cycle at G1/S progression in ASCs exposed to 2 SAR of 1.7 GHz RF-EMF (Fig. 7B). The results in Figs. 6, 7 demonstrated that more cells showed a cell cycle delay at G1/S and SA-β-gal-positive staining when SAR was increased from 1 to 2, supporting our notion that the anti-proliferation effect of RF-EMF was directly correlated with the cell cycle delay and cell senescence. We also confirmed the cell cycle delay and cell senescence with the continuous exposure to 1.7 GHz LTE RF-EMF using well-known molecular markers for G1 to S cell cycle delay. Altogether, these observations strongly demonstrated that the continuous exposure to 1.7 GHz LTE RF-EMF induced cell cycle delay at the G1/S transition and senescence, causing the anti-proliferate effect.

### ROS generated by 1.7 LTE GHz RF-EMF are responsible for the anti-proliferative effect

**Figure 8:**
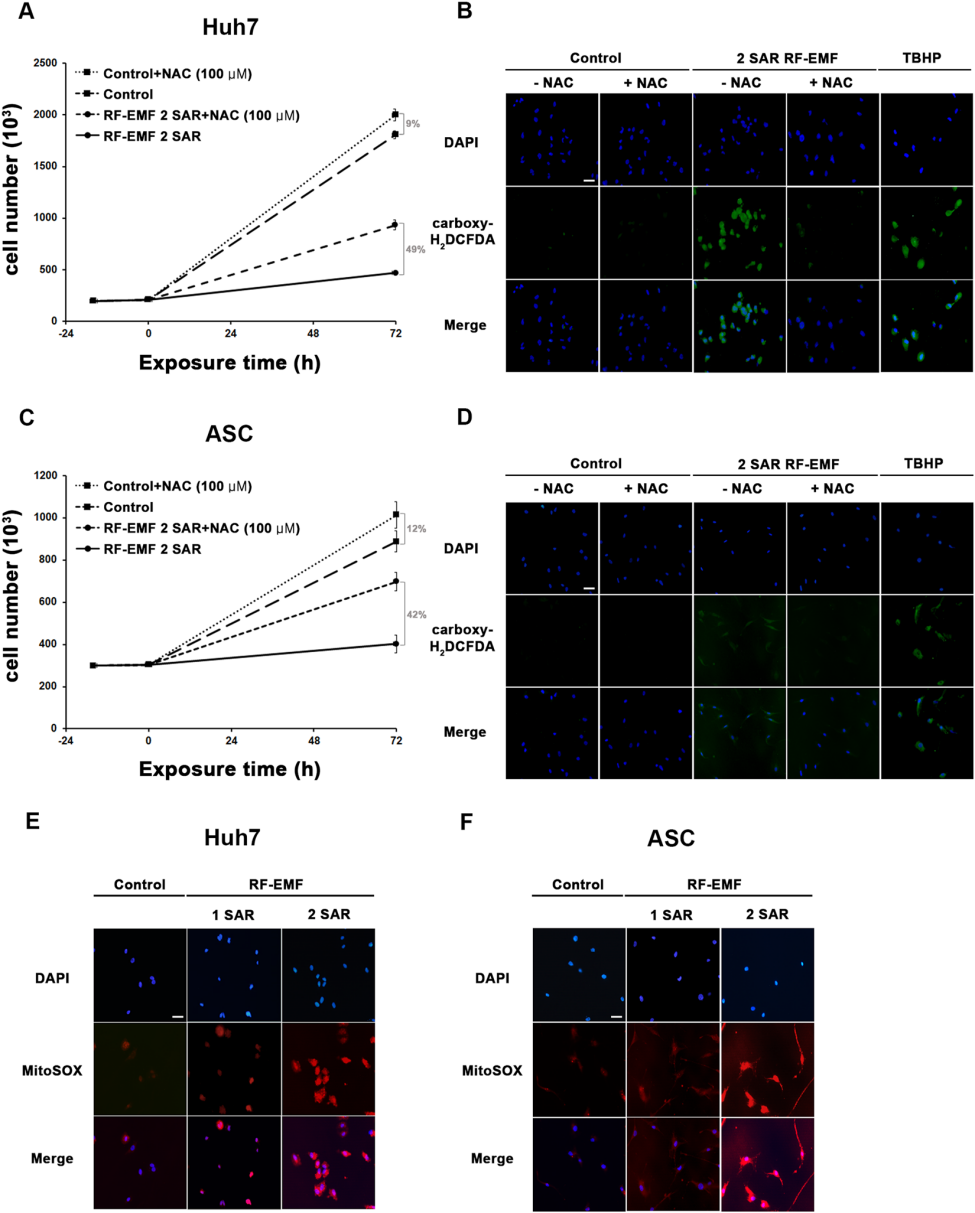
Continuous exposure to 1.7 GHz LTE RF-EMF decreased cell proliferation by inducing intracellular ROS in ASCs and Huh7 cells. (**A–D**) ASCs and Huh7 cells pre-treated or not with 100 μM NAC were exposed to 1.7 GHz RF-EMF for 72 h at 2 SAR, while the sham control cells were incubated for 72 h without RF-EMF exposure. After the exposure, (**A,C**) the cells were collected and counted with a cell counter (Nexcelom Bioscience). Huh7 cells (**B**) and ASCs (**D**) were stained with carboxy-H_2_DCFDA. Cells treat with TBHP were used as a positive control for intracellular ROS generation. (**E,F**) ASCs and Huh7 cells were exposed to 1.7 GHz RF-EMF for 72 h at 1 SAR or 2 SAR, and were stained with MitoSOX. (**B,D–F**) Nuclei were stained with Hoechst 33342. Images were taken with an Axioplan2 fluorescence microscope (Zeiss) under a 200× objective. Scale bar, 25 μm. All experiments consisted of three independent replicates.

As we questioned how 1.7 GHz LTE RF-EMF induced cell senescence, we examined whether intracellular ROS was increased in ASCs and Huh7 cells by 1.7 GHz LTE RF-EMF exposure and whether the decreased proliferation of ASCs and Huh7 cells were due to the ROS generated by RF-EMF. First, we examined the intracellular ROS in ASCs and Huh7 cells exposed to 1.7 GHz LTE RF-EMF with a fluorogenic marker of ROS, carboxy-H_2_DCFDA. Huh7 cells and ASCs treated with tert-butyl hydroperoxide (TBHP) were used as a positive control for increased intracellular ROS generation. When these cells were exposed to 1.7 GHz LTE RF-EMF for 72 h at 2 SAR, at which the anti-proliferative effect was obvious (Fig. 8A,C), both Huh7 cells and ASCs showed increased carboxy-H_2_DCFDA staining compared with the unexposed control cells (Fig. 8B,D). We also observed that carboxy-H_2_DCFDA staining was brighter in Huh7 cells than in ASCs, which is consistent with the stronger anti-proliferative effect in Huh7 cells than in ASCs.

We further confirmed the observation that the continuous exposure to 1.7 GHz LTE RF-EMF increased intracellular ROS in ASCs and Huh7 cells by MitoSOX Red staining. MitoSOX Red allows the selective visualization of O_2_•^−^ generated in the mitochondria, because it is only rapidly oxidized by O_2_•^−^^32^. We could clearly detect bright MitoSOX staining in both RF-EMF-exposed Huh7 and ASC cells and the staining was far brighter in 2 SAR than in 1 SAR (Fig. 8E,F). These observations strongly suggested that 1.7 GHz LTE RF-EMF decreases cell proliferation by increasing intracellular ROS in human cells.

In order to confirm that the decreased proliferation of ASCs and Huh7 cells following the exposure to 2 SAR of 1.7 GHz LTE RF-EMF was attributable to the intracellular ROS generated by the RF-EMF, we compared the viability of ASCs and Huh7 cells with and without 1.7 GHz LTE RF-EMF exposure in the presence and absence of an anti-oxidant. We used N-acetylcysteine (NAC) as an ROS scavenger. If intracellular ROS is responsible for the decreased proliferation, cell viability would be increased in the presence of NAC. As previously reported, treatment with 100 μM NAC increased the proliferation of RF-EMF-unexposed Huh7 cells and ASCs by 9% and 12%, respectively due to its anti-oxidation effect^33,34^. Interestingly, when both Huh7 cells and ASCs were pre-treated with NAC, cell number was increased by 49% in Huh7 and 42% in ASCs following the exposure to 1.7 GHz RF-EMF/LTE for 72 h at 2 SAR, compared with the cells only exposed to RF-EMF without NAC (Fig. 8A,C). These results demonstrated that pre-treatment with the anti-oxidant NAC was far more effective at restoring proliferation in ASCs and Huh 7 cells exposed to 1.7 GHz LTE RF-EMF than at increasing proliferation in the unexposed cells (Fig. 8A,C). Consistent with the restoration of proliferation, intracellular ROS staining was significantly diminished in ASCs and Huh7 cells pre-treated with NAC prior to the exposure to 2 SAR of 1.7 GHz LTE RF-EMF, compared with the cells exposed to RF-EMF without pretreatment (Fig. 8B,D). These results strongly suggested that intracellular ROS generated by 1.7 LTE GHz RF-EMF play a key role in decreasing the proliferation of various human cells, including ASC and Huh7.

## Discussion

Recently 1700 to 1950 MHz RF-EMFs have become the most widely used frequencies in mobile communications and some studies have been carried out to investigate how the exposure to these frequencies of RF-EMF affects living organisms. However, the types and conditions of exposure in the studies were different and the results were not consistent^11,12,21,35,36^. Considering that 1.7 GHz LTE RF-EMF is mainly used in wireless communication, much research has focused on brains or nerves that are exposed to wireless communication equipment^37,38^. However, in order to understand the physiological outcomes and their mechanisms of 1.7 LTE GHz RF-EMF, studies of its effect on various human cells are needed. In this study, we developed an RF-EMF-generating device to minimize the thermal effect and investigated the non-thermal cellular effect of 1.7 GHz LTE RF-EMF on various human cells including human adipose tissue-derived stem cells, liver cancer stem cell populations of Huh7 and Hep3B, HeLa and SH-SY5Y cancer cells, and normal fibroblast IMR-90 cells. We showed that the continuous exposure to 1.7 GHz LTE RF-EMF for 72 h at 1 and 2 SAR decreased cell proliferation in all the cell types studied, regardless of whether they were cancer or normal cells or their tissue of origin. We also showed that the anti-proliferation effects of 1.7 GHz LTE RF-EMF were directly correlated with the SAR value increasing when SAR value was increased from 1 to 2. The RF-EMF-induced anti-proliferative effect was less severe in ASCs, a type of mesenchymal adult stem cells, which is consistent with the well-known observations that stem cells are in general less sensitive to various stresses. One interesting observation was that the neural origin SH-SY5Y cells were the most sensitive among the cell types studied at 2 SAR. Whether neural cells in general are more sensitive to 1.7 GHz LTE RF-EMF than other cell types should be studied further, since brains and nerves are directly exposed to the wireless communication equipment.

We also showed that the anti-proliferative effect of continuous exposure to 1.7 GHz LTE RF-EMF was induced by cell senescence and not by DNA damage and apoptosis. In fact, no obvious DNA DSBs or activation of apoptosis was detected in the exposed cells. Rather, we demonstrated that the decreased cell proliferation induced by exposure to 1.7 GHz LTE RF-EMF was due to a slight cell cycle delay at the G1 to S phase transition and the activation of cell senescence.

The key question was then, how the exposure to 1.7 GHz LTE RF-EMF induces its anti-proliferative effect in human cells. We showed in this study that the exposure to 1.7 GHz LTE RF-EMF increases intracellular ROS and that intracellular ROS generation is directly correlated with SAR; more ROS was generated when SAR increased from 1 to 2. Of note, we detected a significantly enhanced generation of ROS in the mitochondria of cells exposed to 1.7 GHz LTE RF-EMF, suggesting that exposure to RF-EMF affects the efficiency of the electron transport system of mitochondria. Further studies would be needed to understand how RF-EMF affects the electron transport system and the rate of ROS generation in mitochondria.

While increased ROS generation in concert with the exposure to 1.7 GHz LTE RF-EMF in human cells is correlated with the anti-proliferative effect of RF-EMF, it may not be a direct evidence. To verify that the increased ROS is a direct cause of the anti-proliferative effect of RF-EMF, we pre-incubated cells with the anti-oxidant NAC before exposing them to the RF-EMF. We observed that the anti-oxidant could recuperate not only intracellular ROS but also the decreased proliferation, which strongly suggests that ROS are mainly responsible for the anti-proliferative effect of 1.7 GHz LTE RF-EMF.

Several studies that examined the effect of 1.8 GHz RF-EMF, a similar frequency range as we used, have also reported consistent anti-proliferative results as the effect of 1.7 GHz LTE RF-EMF of this study. Ni *et al*. reported that the exposure of human lens epithelial cells to 1.8 GHz RF-EMF at 2, 3, and 4 SAR, each for 6, 12, and 24 h, increased cellular ROS and decreased cellular viability^39^. The exposure to 1.8 GHz RF-EMF at 2 SAR for 24 h caused DNA oxidative damage in the mitochondria and DNA DSB in rat primary neurons^15^. The exposure to 1.8 GHz RF-EMF at 0.15 W/Kg for 3 h also induced a generation of mitochondria ROS and DNA fragmentation in mouse immortalized spermatogonial and spermatocyte cell lines^14^. Altogether, these studies including ours using 1.7~1.8 GHz RF-EMF strongly suggest that the increased ROS generated by RF-EMF induce physiological changes in various mammalian cells, although the physiological outcome would be varied depending on the amount of ROS generated and the sensitivity of different cell types to the specific concentration of ROS. In order to understand the mechanism of cellular ROS generation by RF-EMF, further studies are needed to evaluate the expression and the activity of anti-oxidant enzymes such as glutathione peroxidase, superoxide dismutase, and catalase in cells exposed to 1.7 GHz RF-EMF. The Fenton and Haber-Weiss reaction plays a significant role in oxidative stress by generating •OH (hydroxyl radicals) from hydrogen peroxide (H_2_O_2_) and superoxide (•O2^−^) by intracellular iron ions, serving as a mechanism for cellular aging, because the generated hydroxyl radicals produced by the reaction cause damage to DNA, proteins and lipids in the cell^32^. Depending on the concentration of hydroxyl radicals and the extent of genotoxic damages, physiological responses of cells could be varied ranging from cell cycle arrest and repair to senescence and cell death. To understand the contribution of the Fenton and Haber-Weiss reaction in the observed effect of RF-EMF to induce senescence in this study, the relative changes of intracellular concentrations of hydroxyl radicals over hydrogen peroxide and superoxide by RF-EMF exposure should be accurately measured, although it would be a technical challenge.

Shahbazi-Gahrouel *et al*. examined the effect of GSM mobile phones 900 MHz with intensity of 354.6 μW/cm^2^ on human ASCs, the same type of cells we used in this study, and reported that the proliferation rates of human ASCs were significantly decreased depending on the duration time of exposure^36^. This report showed the consistent anti-proliferative effect of RF-EMF in ASCs with ours, even though two studies used the RF-EMF of different frequencies. On the other hand, Su *et al*. reported that 1.8 GHz RF-EMF exposure to SH-SY5Y cells at 4 SAR for up to 48 h did elicit neither DNA damage nor abnormal cellular behaviors^9^. This result is consistent with ours in that no DNA damage was induced by 1.7~ 1.8 RF-EMF, but different from ours regarding its effect on cell proliferation. Since the exposure time in our study was 72 h while it was 42 h in the study of Su *et al*., we predict that a continuous exposure span might be a key factor to produce different physiological outcomes of human cells by RF-EMF.

Altogether, this study as well as other studies strongly suggest that RF-EMF exposure leads to a change in intracellular ROS levels that may result in genotoxic stress, decreased proliferation and cell senescence, or no physiological effects depending on ROS concentration and the differential sensitivity of various cells to ROS. Thus, the mechanism behind RF-EMF exposure altering intracellular ROS levels should be further studied to elucidate the biological effects of RF-EMFs.

It is not plausible to directly predict the physiological effects of 1.7 GHz LTE RF-EMF from our cell-based study. However, the anti-proliferative effect of 1.7 GHz LTE RF-EMF on various human cells in this study suggests that the exposure to 1.7 GHz LTE RF-EMF would be more harmful to children, whose adult stem cells should be very active for growth and may accelerate the aging of body cells. We also carefully suggest that the anti-proliferative effect of various cancer cells by 1.7 GHz LTE RF-EMF would be interpreted with care, considering that both positive and negative effects of RF-EMF have been reported on cancer development.

## Materials and Methods

### Design of the radio frequency cell exposure system

A radial transmission line (RTL) exposure system^40–42^ was used as the *in vitro* exposure system, because they can simultaneously expose a large number of culture dishes. The 1.76 GHz LTE signal was applied to the RTL exposure system (Fig. 1A). The exposure level and schedule were controlled by a control unit and the maximum input power was 60 W. The input signal was fed through a conical antenna with broadband characteristics. The external dimensions of the exposure system were 843 mm × 825 mm × 315 mm (Fig. 1B). The exposure system was specifically designed to control environmental conditions including ventilation, humidity, and temperature. To maintain CO_2_ density and humidity inside the chamber, the gas from an incubator was circulated throughout the chamber. Additionally, a water pump circulating water throughout the bottom of the cavity was used to control temperature. In this study, two types of water pumps were used. One was used to circulate the water at the set temperature without engaging the cooling system, and the other was used to protect against rising culture medium temperature during RF exposure by circulating the water from the cooling system. The return loss of the exposure system was under -10 dB from 800 MHz to 5 GHz (Fig. 1C), which is the ratio of the reflected power to the incident power in decibels (dB). It means how well the system matches without reflection. The system works well if the return loss is under -10 dB at operation frequency. The SAR measurement was performed using a Luxtron 812 fiber optic thermometer from Luxtron Corporation with a thermal resolution of 0.1 °C. The measurement probes were located at nine points inside a Petri dish (Fig. 1D). Figure 1E shows the graphs of the cell culture temperature at the center of a Petri dish for 1.7 GHz LTE exposure. The slope of the temperature rise is derived by linear fitting from the measured temperatures. The SAR values were calculated from the temperature rise using the equation given below:

$${\rm{SAR}}={{\rm{C}}}_{{\rm{p}}}\frac{\varDelta {\rm{T}}}{\varDelta {\rm{t}}}\approx {{\rm{C}}}_{{\rm{p}}}\frac{{\rm{dT}}}{{\rm{dt}}}$$

where C_p_, T, and t are the specific heat (J/kg °C), temperature rise by exposure (°C), and exposure time (s), respectively. Three measurements were carried out at nine points, as shown in Fig. 1D. The mean value and standard deviation of the SAR measurements for the entire sample were 0.155 ± 0.004 W/kg/W, which was normalized to the input power of 1 W at the feeding point.

To examine the biological effects of the RF field through *in vitro* cell experiments, the device was installed in an incubator that provides an environment similar to that in the body (Fig. 2C). To maintain constant temperature of the incubator, a water cooling system was installed to suppress the increased temperature by the electromagnetic fields (Fig. 2B). The electromagnetic field exposure device has an antenna at its center, which generates an electromagnetic field and the culture dishes were placed a slight distance away from the center of the device. We had to set the exact field strength to expose the cells at the specific absorption rate (SAR) (Fig. 2D,E). To expose the cells with the desired SAR, a SAR conversion table was prepared using engineering calculations (Fig. 2F). As shown in Fig. 2G, the SAR value (upper figure) as well as the temperature of the incubator (lower, yellow line), and the temperature of a refrigerated water-cooling system (lower, red line) of the RF-EMF exposure device was continuously monitored during cell exposure experiments.

### Sources of cells and cell culture

Human ASCs were purchased from Thermo Fisher scientific. HeLa and IMR90 cells were purchased from the American Type Culture Collection (ATCC). Stem cell populations of human hepatocellular carcinoma Huh7 and Hep3B were gifts from Dr. Y. N. Park (Yonsei University College of Medicine, Seoul, Korea). The human neuroblastoma SH-SY5Y was kindly provided by Dr. Inhee Mook-Jung (Seoul National University College of Medicine, Seoul, Korea). The characteristics of ASC, Huh7, and Hep3B were confirmed by checking the specific cell markers by RT-PCR (Supplementary Fig. S1) and primers used for RT-PCR were shown in Table 1.

**Table 1:** Sequences of the primers used for RT-PCR in this study.

**CD44**	Forward	GATCCACCCCAACTCCATCT
	Reverse	AACTGCAAGAATCAAAGCCA
**CD105**	Forward	TGTCTCACTTCATGCCTCCAGCT
	Reverse	AGGCTGTCCATGTTGAGGCGAT
**CD45**	Forward	ACCAGGGGTTGAAAAGTTTCAG
	Reverse	GGGATTCCAGGTAATTACTCC
**FABP4**	Forward	ACTGGGCCAGGAATTTGACG
	Reverse	CTCGTGGAAGTGACGCCTT
**CD133**	Forward	CAAGATACTTCAACGCACAGG
	Reverse	CATCGTACACGTCCTCCGAA
**EpCAM**	Forward	AATCGTCAATGCCAGTGTACTT
	Reverse	TCTCATCGCAGTCAGGATCATAA
**ALDH1A**	Forward	TACCTGTCCTACTCACCGAT
	Reverse	GATCTTGTCAGCCCAACCT
**ACTIN**	Forward	TCCCTGGAGAAGAGCTACGA
	Reverse	AGCACTGTGTTGGCGTACAG

Human ASCs were cultured in Dulbecco’s modified Eagle’s medium/F12 (DMEM/F12, Gibco/Invitrogen, Grand Island, NY, USA), human hepatocellular carcinoma Huh7 and Hep3B, human neuroblastoma SH-SY5Y, and HeLa were cultured in DMEM (Gibco/Invitrogen, Grand Island, NY, USA), both mediums were supplemented with 10% (v/v) fetal bovine serum (FBS; Sigma-Aldrich, MO, USA) and 10 mL/L penicillin-streptomycin (GIBCO, NY, USA). IMR90 was maintained in minimum essential media (MEM; Gibco/Invitrogen, Grand Island, NY, USA) with 10% (v/v) fetal bovine serum (FBS; GIBCO, NY, USA) and 10 mL/L penicillin-streptomycin (GIBCO, NY, USA). All cells were grown at 37 °C in a humidified atmosphere containing 5% CO_2_.

### Cell exposure with the LTE RF-EMF radiation system

The exposure system was warmed up for at least 30 min, for equilibration, before RF-EMF exposure. A total of 30 × 10^4^ ASC cells and 20 × 10^4^ Huh7, Hep3B, HeLa, SH-SY5Y, and IMR 90 cells were seeded and incubated in 100 mm dish for 16 h before RF-EMF exposure. The 100-mm culture dishes were placed 13.6 cm from the conical antenna, which was located at the center of the exposure chamber. Cells in the culture dishes were then continuously exposed to the RF-EMF radiation of a single LTE signal (WCDMA signal at 1700 MHz) at 1 W/kg or 2 W/kg for 72 h. During the exposure, the temperature of the incubator was maintained at 35.5 ± 0.5 °C by circulating water within the cavity, and a 5% CO_2_ concentration was also maintained in the chamber. For both cells for RF-EMF exposure and the sham untreated control, the same cells were seeded at the same time and incubated in a 100 mm dish for 16 h before RF-EMF exposure in the same incubator. The untreated sham group was incubated for 72 h in the same kind of incubator under the same incubation condition as the incubator connected to the RF-EMF device. After 72 h, the number of cells in each dish of the RF-EMF-exposed and the sham group was counted using a Cellometer Auto T4 (Nexcelom).

### Western blot analysis

RF-EMF/LTE-exposed cells were harvested and lysed with a lysis buffer as previously described^43^. Histones were extracted with 0.2 M HCl and neutralized with 1 M NaOH. Cell lysates or histones were separated on a 8–10% SDS-polyacrylamide gel (PAGE), transferred to a PVDF membrane (Merck Millipore, Billerica, MA, USA), and detected using the following primary antibodies: anti-poly ADP-ribose polymerase (PARP; Cell Signaling Technology, Inc., MA, USA), anti-phospho-H2AX (γ-H2AX; Millipore, Germany), anti-p21 (Santa Cruz Biotechnology, Inc., CA, USA), anti-p53 (Santa Cruz Biotechnology, Inc., CA, USA), anti-phospho-p53 (Cell Signaling Technology, Inc., MA, USA), anti-retinoblastoma (Rb; Cell Signaling Technology, Inc., MA, USA), and anti-phospho-Rb (Cell Signaling Technology, Inc., MA, USA), anti-actin (Cell Signaling Technology, Inc., MA, USA), and anti-histone3 (Cell Signaling Technology, Inc., MA, USA). An enhanced chemiluminescence system (Amersham Biosciences) was used to visualize the blots.

### Senescence-associated β-galactosidase activity assay

Senescence-associated β-galactosidase (SA-β Gal) staining was conducted on ASCs and a CSC population of Huh7 cells exposed to RF-EMF/LTE for 72 h. After incubation for 72 h in RF-EMF/LTE, the cells were fixed in 2% formaldehyde and 0.2% glutaraldehyde for 15 min at room temperature, followed by incubation with an X-gal (Sigma-Aldrich, MO, USA) solution for 30 h, as described previously^34^. For positive senescence controls, ASCs were treated with H_2_O_2_ 200 μM for 1 h and Huh7 was treated with H_2_O_2_ 300 μM for 2 h. SA-β Gal-stained cells were observed with a Nikon microscope (ECLIPSE Ts2) under a 10× objective and monitored with HKBasic (http://www.koptic.co.kr).

### Flow cytometry analysis

RE-EMF/LTE-exposed cells were harvested using 0.25% trypsin–EDTA (Gibco-BRL), and washed with warm PBS. Cells were fixed in 70% ethanol for 1 h at 4 °C, treated with RNase A (100 μg/ml) for 30 min at 37 °C, and stained with propidium iodide (PI 50 μg/ml) for 1 h at 4 °C in the dark. After staining, flow cytometry was performed using FACSCalibur (BD Bioscience) and analyzed with Flowing (http://flowingsoftware.btk.fi).

### Detection of intracellular ROS

After being seeded and incubated for 16 h, cells were cultured in fresh medium containing 100 μM N-acetyl-L-cysteine (NAC) (Sigma-Aldrich, MO, USA) with continuous RF-EMF exposure for 72 h. ASC and Huh7 cells were independently incubated with tert-Butyl hydroperoxide (TBHP) to generate intracellular ROS as positive controls. Intracellular ROS were measured using an ROS detection kit (Invitrogen, CA, USA) following manufacturer’s protocol, as previously described^31^. Briefly, the cells were washed with PBS and stained with 25 μM 5-(and-6) carboxy-2′,7′-dichlorodihydrofluorescein diacetate (carboxy-H_2_DCFDA) for 30 min at 37 °C. 1 μM Hoechst 33342 was added to the carboxy-H_2_DCFDA staining solution for the last 5 min of incubation. Cells were fixed and observed with Axioplan2 fluorescence microscopy (Zeiss) with a 200× objective.

In order to detect mitochondrial ROS, ASCs and Huh7 cells were washed twice with warm PBS and incubated with 5 μM MitoSOX Red (Thermo Fisher Scientific) in PBS for 20 min at 37 °C in the dark. 1 μM Hoechst 33342 was added to the MitoSOX Red solution for the last 5 min of incubation. After treatment, the cells were washed three times with warm PBS, fixed and observed with Axioplan2 fluorescence microscopy (Zeiss) with a 200× objective.

### Chemical regent sources

Dulbecco’s Modified Eagle’s Medium/F-12 (DMEM/F12), DMEM, minimal essential media are purchased from Gibco/Invitrogen (Grand Island, NY, USA). The X-gal and N-acetyl-L-cysteine (NAC) were purchased from Sigma-Aldrich (Saint Louis, MO, USA). The ROS detection kit was purchased from Invitrogen (Carlsbad, CA, USA). The MitoSOX Red was purchased from Thermo Fisher Scientific (Hillsboro, OR, USA).

### Statistical analysis

All statistical analyses were performed using GraphPad Prism 5 (GraphPad Software, Inc., CA, USA). All data are presented with the mean ± standard deviation (S.D.) of more than three independent experiments with statistical significances. P < 0.05 (*), P < 0.01(**), and P < 0.001(***) were considered statically significant, and P > 0.05 was considered statically non-significant (ns).

## Supplementary information

Supplementary information.
