# Supplementary material for: Continuous Exposure to 1.7 GHz LTE Electromagnetic Fields Increases Intracellular Reactive Oxygen Species to Decrease Human Cell Proliferation and Induce Senescence
Source: Sci Rep. 2020 Jun 8;10:9238. doi: 10.1038/s41598-020-65732-4 (PMC7280220; doi:10.1038/s41598-020-65732-4)
Supplement: Supplementary file 1 — Supplementary information. [file 41598_2020_65732_MOESM1_ESM.docx]

**Supplementary Information**

**Continuous Exposure to 1.7 GHz LTE Electromagnetic Fields Increases Intracellular Reactive Oxygen Species to Decrease Human Cell Proliferation and Induce Senescence**

Jisu Choi^1,**^, Kyeongrae Min^1,**^, Sangbong Jeon ^2^, Nam Kim^3^, Jeong-Ki Pack^4^, and Kiwon Song^1, *^

^1^Department of Biochemistry, College of Life Science & Biotechnology, Yonsei University, Seoul 03722, Korea

^2^Radio & Satellite Research Division, Broadcasting·Media Research Laboratory, Electronics and Telecommunications Research Institute (ETRI), Daejon 34129, Korea

^3^School of Information and Communication Engineering, Chungbuk National University, Cheongju, Chungbuk 28644, Korea

^4^Department of Radio and Information Communications Engineering, Chungnam National University, Daejon 34134, Korea

*Address correspondence to Kiwon Song, Department of Biochemistry, College of Life Science and Biotechnology, Yonsei University, Seoul 120-749, Korea

Tel.: 82-2-2123-2705, Fax: 82-2-362-9897, E-mail: [bc5012@yonsei.ac.kr](mailto:bc5012@yonsei.ac.kr)

** Both authors contributed equally to this work.


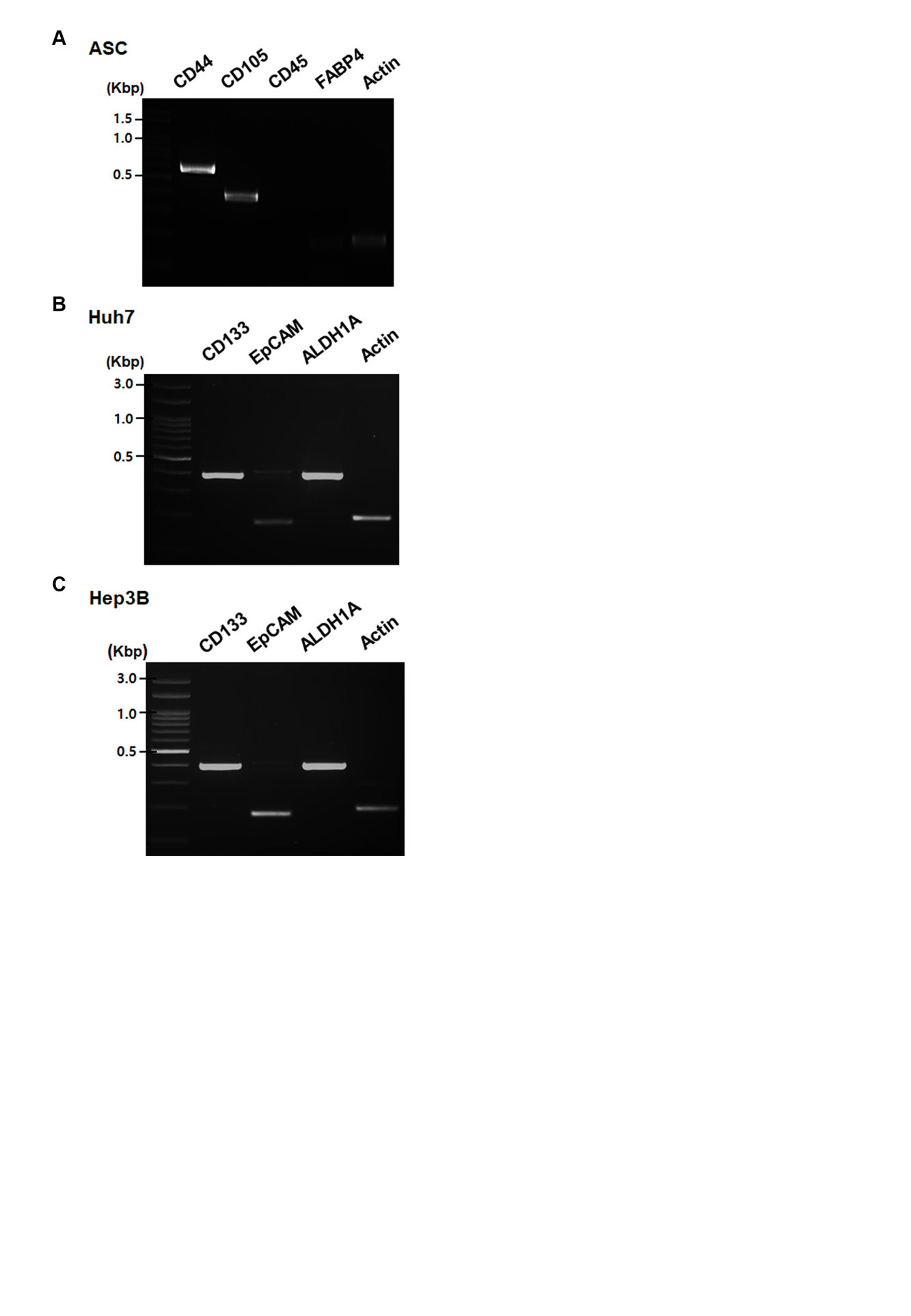


**Supplementary Figure S1. Verification of the cell markers for ASCs, and Huh7 and Hep3B cancer stem cells used in this study**

Stem cell characteristics of ASC, Huh7, and Hep3B were confirmed by identifying the expression of cell markers. (A-C) ASC, Huh7, and Hep3B cells were harvested at 80% confluence and total RNA was extracted with an RNasey Mini kit (QIAGEN). Purity of the extracted RNA was assessed by electrophoresis on a 0.8% agarose gel and cDNA was synthesized using a Biotechnology Power cDNA Synthesis Kit (2511; iNtRON). The PCR reaction was performed with the synthesized cDNA using an i-MAX DNA polymerase kit (iNtRON) along with primers specific for CD44, CD105, CD45, and fatty acid binding protein 4 (FABP4) in ASC, and CD133, EpCAM, aldehyde dehydrogenase 1A (ALDH1A) in Huh7 and Hep3B. β-actin was used as a positive control for cDNA synthesis and PCR. The PCR products were analyzed by electrophoresis on a 2.5% agarose gel. The primers used for PCR were shown in the Table 1. (A) CD44 and CD105 were used as positive markers, and CD45 as a negative marker for ASCs. FABP4 was also used as a marker of differentiation into adipocytes. (B, C) CD133 and EpCAM were used as cancer stem cell markers and ALDH1 was used as a hepatocyte marker. Samples were analyzed in 2.5 % agarose gel electrophoresis. Actin was used as an RNA expression marker in each experiment.


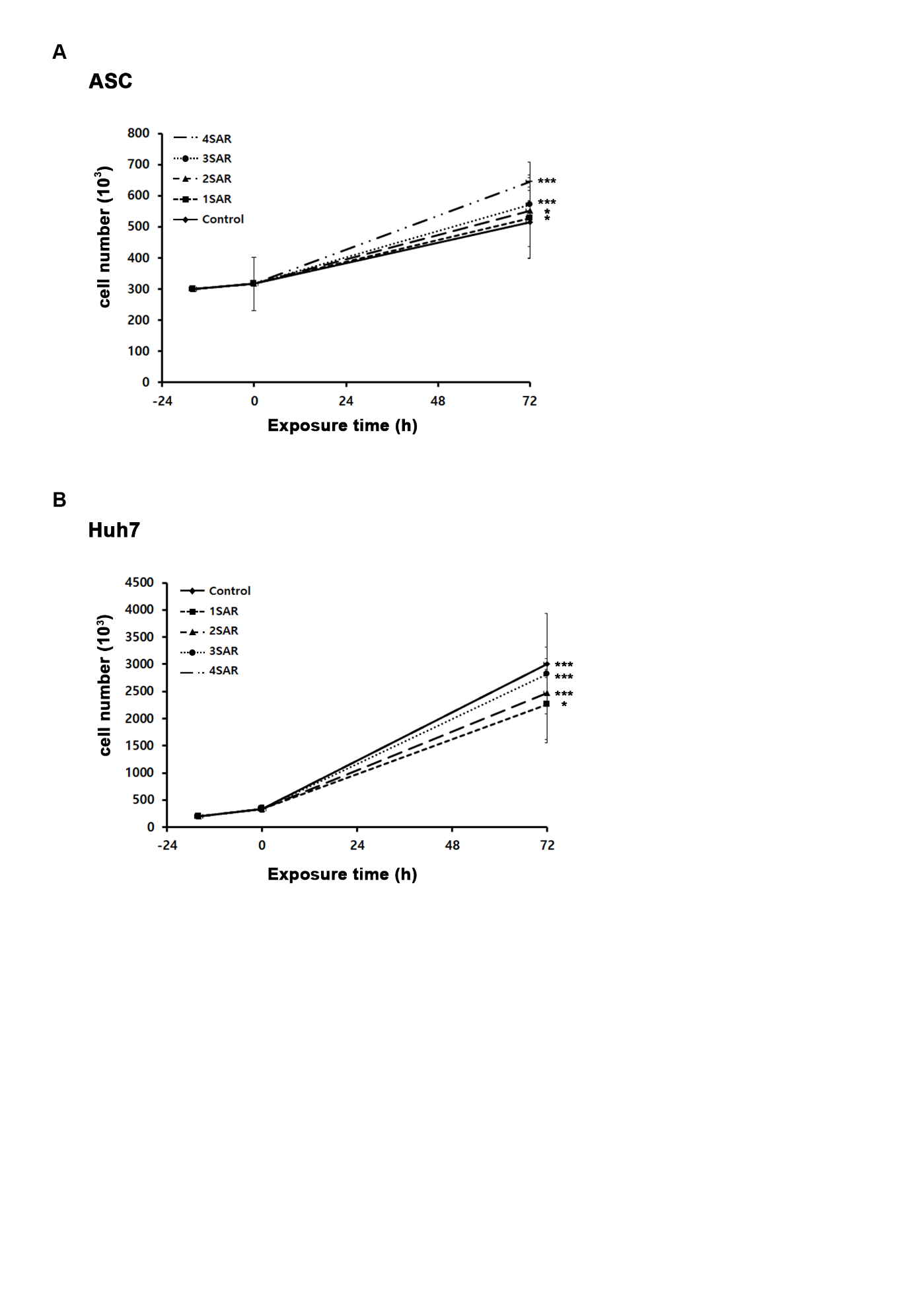


**Supplementary Figure S2. The thermal effect of 1.7 GHz LTE RF-EMF without a forceful cooling system activated cell proliferation**

The same number of (A) ASCs and (B) Huh7 cells prepared as described in Materials and Method was respectively exposed to 1.7 GHz LTE RF-EMF for 72 h at 1, 2, 3, and 4 SAR. For RF-EMF exposures, the same device was used without a water-cooling system for the incubator to forcibly lower the heated water temperature by RF-EMF. The sham control cells were incubated for 72 h without RF-EMF exposure. After each exposure, cells were collected and counted with a cell counter (Nexcelom Bioscience). At least three independent experiments were performed and the cell number was plotted as mean ± S.D. P < 0.01(**), P < 0.001(***), and P > 0.05 (non-significant, n.s.).

When the temperature of the incubator without a forceful water-cooling system was measured, it was gradually increased from 37°C to 39°C with increasing SAR values from 1 to 4. As reported that the slight increase of incubating temperature usually activates the proliferation of mamalian cells [1], we could detect the increased proliferation in ASCs and Huh7 with increasing SAR values, compared with their proliferations in the RF-EMF at the same SAR value with a forceful cooling system. We overall observed the slight increase of proliferation in ASCs and the decrease of proliferation in Huh7, which would be the combined effects of temperature and RF-EMF.

[1] Y.M. Choi, P.R. Chen, S. Shin, J. Zhang, S. Hwang, K. Lee, Mild heat stress enhances differentiation and proliferation of Japanese quail myoblasts and enhances slow muscle fiber characteristics, Poult Sci 95(8) (2016) 1912-7.


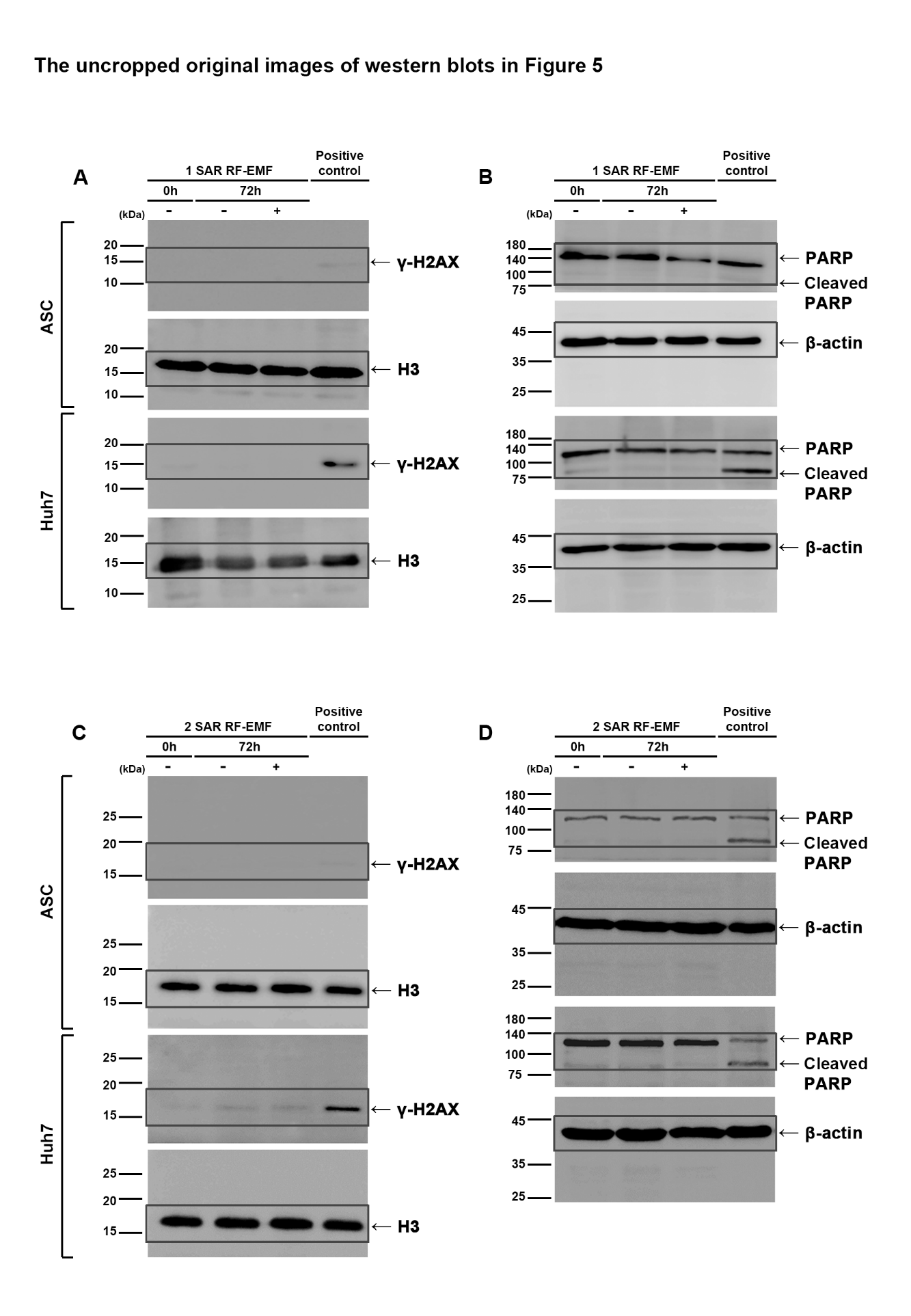


**Supplementary Figure S3. The uncropped original images of western blots in Figure 5**

Cell lysates or histones were separated on a 8-10% SDS-polyacrylamide gel (PAGE), transferred to a PVDF membrane (Merck Millipore, Billerica, MA, USA), and detected using primary antibodies for the protein of interest and for its proper loading control. (A, C) To detect γ-H2AX and its loading control H3, histone preparation was separated on a 10% SDS-PAGE, transferred to a membrane, and incubated with anti-γ-H2AX. The same blot was stripped and re-probed with anti-H3 as a loading control. (B, D) Cell lysate was separated on a 8% gel, transferred to a membrane, and cut into two pieces to be developed with anti-PARP and with β-actin as a loading control from the same gel. The complete scanned images for western blots shown in Figure 5 with MW markers. Black boxes are the cropped parts shown in each respective figure.


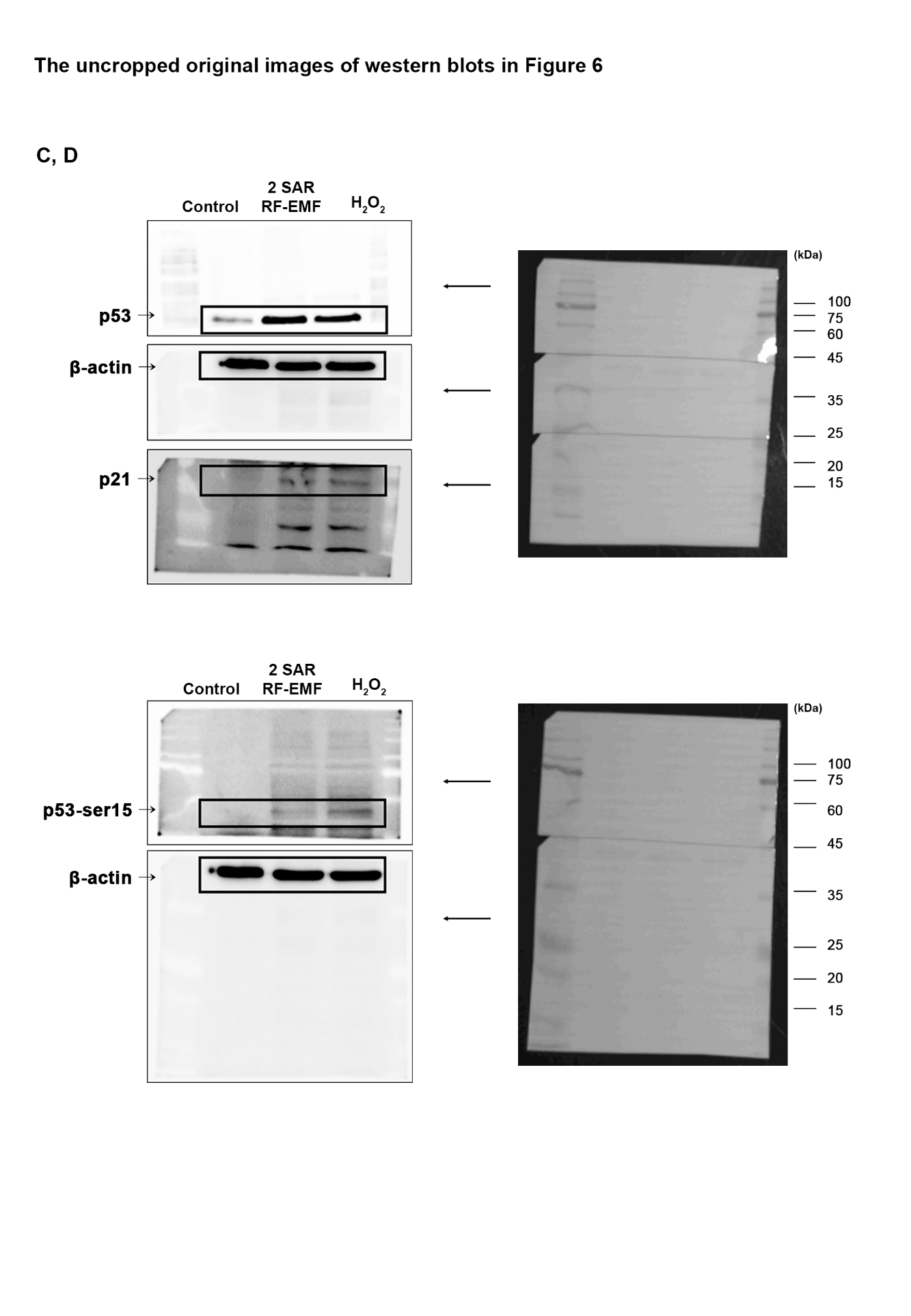


**
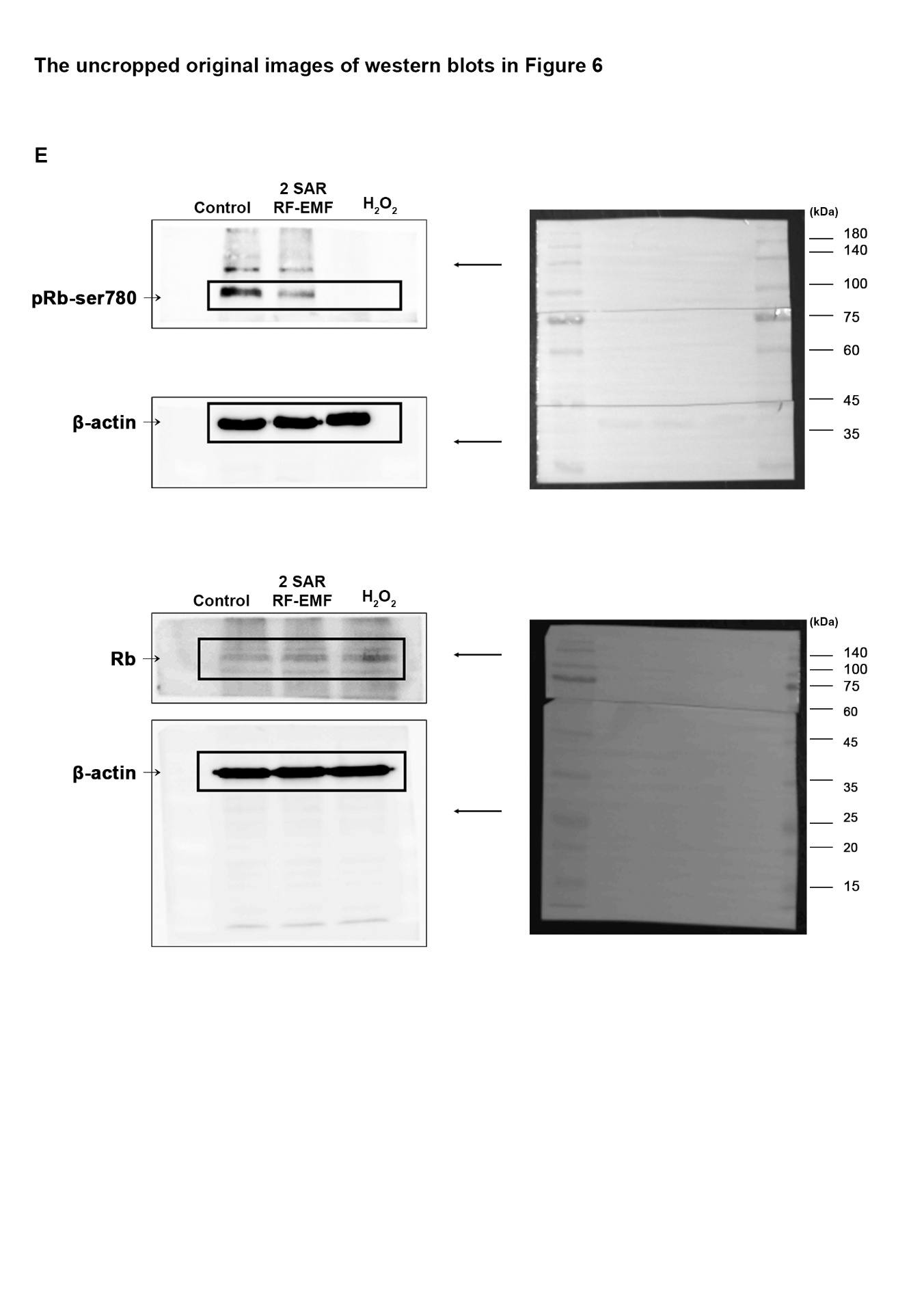
**

**Supplementary Figure S4. The uncropped original images of western blots in Figure 6**

Cell lysates were separated on a 10% SDS-polyacrylamide gel (PAGE), transferred to a PVDF membrane (Merck Millipore, Billerica, MA, USA), and cut into two or three pieces to detect the protein(s) of interest along with a loading control β-actin in the same gel. The complete scanned images for the pieces of the original blot taken together from the same gel (right panels, cut into two or three pieces before incubation with antibodies of interest and a loading control β-actin). Their western blots (left panels) were shown in Figure 6 C-E with MW markers. Black boxes are the cropped parts shown in each respective figure.
